# Beneficial Effects of Elderly Tailored Mediterranean Diet on the Proteasomal Proteolysis

**DOI:** 10.3389/fphys.2018.00457

**Published:** 2018-05-01

**Authors:** Sophia Athanasopoulou, Niki Chondrogianni, Aurelia Santoro, Konstantina Asimaki, Vasiliki Delitsikou, Konstantinos Voutetakis, Cristina Fabbri, Barbara Pietruszka, Joanna Kaluza, Claudio Franceschi, Efstathios S. Gonos

**Affiliations:** ^1^Institute of Biology, Medicinal Chemistry and Biotechnology, National Hellenic Research Foundation, Athens, Greece; ^2^Department of Experimental, Diagnostic and Specialty Medicine, University of Bologna, Bologna, Italy; ^3^C.I.G. Interdepartmental Centre “L. Galvani”, University of Bologna, Bologna, Italy; ^4^Department of Human Nutrition, Warsaw University of Life Sciences – SGGW, Warsaw, Poland; ^5^Institute of Neurological Sciences (IRCCS), Bologna, Italy

**Keywords:** proteolysis, nutrition, aging, proteasome, frailty, inflammation, anti-oxidants

## Abstract

Aging is a multifactorial process characterized by the accumulation of proteins undergoing oxidative modifications, either due to enhanced levels of oxidative stress or due to their decreased clearance; both facts are related to the establishment of chronic inflammatory processes. These processes are directly associated with functional and structural modifications of a key cellular component, namely the proteasome. In this study, levels of oxidized proteins, along with proteasome and immunoproteasome composition and activity on a selected group of 120 elderly volunteers were analyzed before and after the administration of a specific dietary protocol, based on an elderly tailored Mediterranean diet (the “NU-AGE diet”). A significant negative correlation between levels of oxidized/carbonylated proteins and proteasome function was confirmed, both before and after intervention. Furthermore, it was demonstrated that subgroups of non-frail subjects and women receive a greater benefit after the intervention, concerning specifically the proteasome content and activity. These data highlight the putative beneficial effects of Mediterranean diet on the major cellular proteolytic mechanism, the proteasome, in elderly people.

## Introduction

With a continuously aging population, the impact of chronic diseases and dependencies, for which no specific cures are available, increases dramatically. This transitional social phenomenon, that affects individuals and societies, poses a real threat for current health care systems worldwide. Unfortunately, in a number of countries, healthy life expectancy appears not to follow the same increasing trend as life expectancy. For instance, within the EU, whereas it has been described that human life expectancy by the age of 50 has risen by 1.2 years between 2005 and 2010, healthy life expectancy for the same period of time has only increased slightly (Fouweather et al., 2015). This condition is favored by phenomena such as social and economic crises, that lead to increased inequalities between these countries in healthy lifespan. Within this frame, EU action plans would inevitably recruit and encompass scientific research strategies that would be focused in the improvement of the quality of life in old age. In support, a wide gamut of epidemiological data has elucidated the beneficial role of specific dietary interventions in combatting and delaying the aging process and accompanying pathologies, such as age-related diseases, frailty, disability, morbidity and mortality, both in animal models and humans. Those pathologies have been largely associated with a physiological phenomenon, characterized by the establishment of a chronic and low grade inflammatory status in the elderly, so called “inflammaging” (Franceschi et al., 2000, 2007; Cevenini et al., 2013). Mediterranean Diet (MedDiet) has been extensively investigated by several observational, longitudinal and randomized-controlled studies for its pivotal role in the prevention of a wide range of chronic age-related pathological conditions and the reduction of all causes of mortality (Korre et al., 2014). In particular, the traditional MedDiet, a cultural heritage of humanity, characterized by consumption of non-starchy vegetables, fruits, cereals, legumes, cold pressed olive oil, low intake of meat, fish, and dairy products and a moderate consumption of alcohol, provides a balanced combination of nutrients that have antioxidant, anti-inflammatory and prebiotic activity, and also elicits an integrated network of cellular mechanisms of stress/oxidative damage response (Martucci et al., 2017). Numerous epidemiological studies in populations showing adherence to MedDiet have indicated it’s protective effect against stroke, cardiovascular disease, obesity, neurodegenerative disorders, cancer, hypertension, diabetes, and allergic reactions (Chatzianagnostou et al., 2015). Although the exact underpinning mechanism by which the traditional MedDiet lowers the risk of certain cancers, metabolic and cardiovascular disorders has not been fully depicted, however, many potential mechanisms have been proposed as the major mediators of its beneficial effects. Those encompass its lipid lowering effect, combatting oxidative stress, along with inflammation and platelet aggregation, metabolic regulation of different molecular pathways associated with tumorigenesis, tumor progression, nutrient- sensing and gut-microbiota (Tosti et al., 2018). Certain phytochemicals typically found in MedDiet, such as lignans, ferulic acid, spermidine, apigenin, phytic acid, etc., encountered essentially in whole grains and olive oil, have been proved to possess anti- inflammatory and anti-oxidant potential via reducing inflammation and cell necrosis during aging, increasing autophagy and regulating ER stress modulators such as histone acetyltransferases, the inhibition of which leads to higher resistance to oxidative stress (Tosti et al., 2018). To this end the European project (NU-AGE) was established to test the possibility to reduce inflammaging through a newly designed, personally tailored Mediterranean dietary pattern (MedDiet), designed to meet the nutritional needs of people over 65 years of age (NU-AGE diet) (Berendsen et al., 2014). The NU-AGE project was also aimed to unravel the cellular and molecular pathways that are affected by the MedDiet by a comprehensive monitoring of a variety of domains using an integrated classical, *-omic* and systems biology approach (Santoro et al., 2014).

One aspect that has emerged as an important factor associated with effective counteracting of inflammaging is the maintenance of proteostasis. The proteasome, being the main proteolytic cellular system, plays a crucial role in maintaining proteostasis (Chondrogianni et al., 2014). Specifically, the UPS is involved in the regulated degradation of non-functional or excessive proteins and participates in numerous and diverse cellular functions. The 20S proteasome is a barrel-like structure composed of 28 protein subunits that form a complex of 700 kDa. The two outer rings comprise seven different α subunits, while the interior rings consist of seven β subunits, creating a α1-7/β1-7/β1-7/α1-7 layout. The external α rings control the entry of proteasome’s substrates into the β rings, where the proteolytic activity is held. The α-subunits additionally bind different regulatory factors related with the activity and specificity of the catalytic core. β1, β2, and β5 out of the 7 β subunits show proteolytic activity, bearing different substrate specificity. Specifically, β1 has a caspase-like activity (CL or PGPH), β2 a trypsin-like (TL) and β5 a CT-L. The protein hydrolysis occurs after acidic peptide bonds, basic amino acids and hydrophobic amino acids, respectively. Professional antigen-presenting cells (APCs, such as dendritic cells), a specific cell population capable of triggering T- cell activation, express an alternative form of proteasome in which the catalytic β1, β2, and β5 subunits are replaced with β1i (LMP2), β2i (LMP10), and β5i (LMP7), respectively (Chondrogianni et al., 2015a). Immunoproteasomes are considered to be related with enhanced antigen presentation and processing, as they are constitutively expressed in APCs, but also because two of the aforementioned immunosubunits are encoded by genes located on the same chromosome with genes encoding major histocompatibility complex (MHC) class II and TAP (Zanker and Chen, 2014). Importantly, it has been demonstrated that the accumulation of damaged proteins during aging is linked to an age-related decline in proteasome content and activities, which in turn, is due to the down-regulation of the catalytic subunits of the 20S complex (Chondrogianni et al., 2003). Stable over-expression of proteasome’s catalytic subunits restores proteasome activities and function, while the “proteasome activated” human cell lines exhibited a significant delay of senescence (Chondrogianni et al., 2005). Recent findings also propose that proteasome activation is an evolutionary conserved mechanism, as it can delay aging *in vivo* and it also confers deceleration of aggregation-related pathologies, such as Alzheimer’s or Huntington’s diseases (Chondrogianni et al., 2015b; Papaevgeniou et al., 2016). Therefore, given these findings we examined in this study whether proteasome levels and activity as well as levels of oxidized/carbonylated proteins and immunoproteasome levels were affected by implementation of the aforementioned dietary intervention.

## Materials and Methods

### Study Design and Samples Selection

The NU-AGE study is a 1-year, randomized, parallel trial carried out in five European study centers (Bologna, Italy; Norwich, United Kingdom; Wageningen, Netherlands; Warsaw, Poland; and Clermont-Ferrand, France). Recruitment started in April 2012 and finished in January 2014 including 1,296 apparently healthy European men and women aged 65–79 years. The rationale and design of this intervention study are described in detail elsewhere (Berendsen et al., 2014; Santoro et al., 2014). In short, participants completed questionnaires about their health and lifestyle and a 7-day food record to obtain information about their dietary intake. Tests were conducted to measure a wide range of health outcomes. All questionnaires and measurements were repeated after 1 year. Half of the participants were randomly assigned to the intervention arm (NU-AGE diet), while the remaining fifty percent were controls (they continued to follow their habitual dietary habits). The study protocol was approved by local medical ethics committees at all study sites and the NU-AGE study is registered with clinicialtrials.gov since December 21st 2012 (NCT01754012). The study protocol is approved by the South-East 6 Person Protection Committee (France), Independent Ethics Committee of the S. Orsola-Malpighi Hospital Bologna (Italy), the Wageningen University Medical Ethics Committee (Netherlands), the National Research Ethics Committee – East of England (United Kingdom) and the Bioethics Committee of the Polish National Food and Nutrition Institute (Poland). All subjects gave written informed consent in accordance with the Declaration of Helsinki.

From the whole NU-AGE cohort, a sub-group 120 subjects at T0 and T1 (after dietary intervention) has been randomly selected from the Italian and Polish sub-cohorts to be analyzed by specific -omics and proteasome composition and activity. This sub-group included half pre-frail and half non-frail subjects matched per gender and age, belonging to the intervention arm. Frailty was based on the five criteria proposed by Fried et al. (2001) including weight loss, weakness (i.e., poor handgrip strength), self-reported exhaustion, slowness (i.e., slow gait speed), and low physical activity. Weight loss was defined as self-reported unintentional loss (i.e., not due to diet or physical exercise) of ≥4.5 kg in the last 12 months. Handgrip strength was measured three times in the dominant hand using the Scandidact Smedley’s Hand Dynamometer^®^. Weakness was defined as the average handgrip strength equal or below the sex- and BMI-specific cutoffs provided by Fried et al. (2001). Two questions from the Center for Epidemiologic Studies Depression (CES-D) scale were administered as measures of exhaustion: “I felt that everything I did was an effort” and “I could not get going.” Self-reported exhaustion was present if at least one condition was present for ≥3 days in the past week. Gait speed was measured by asking participants to walk at their usual speed over 4.5 m. Slow gait was defined as walking equal to or above the sex- and height-specific validated cut-offs (Fried et al., 2001). Physical activity was measured with the energy expenditure weekly rate (kcal/week) derived from the modified Minnesota Leisure Time Activity Questionnaire, in which participants were asked on frequency and duration of time spent in 18 activities over the prior 2 weeks (Taylor, 1978; Siscovick et al., 1997). Low physical activity was defined as <383 kcal for men or <270 kcal for women, according to sex-specific cutoffs (Fried et al., 2001). The absence of any of the above criteria defines non-frailty status, while pre-frailty was defined as the presence of 1 or 2 of the above criteria.

### Blood Handling and PBMC Collection

Blood samples have been collected with ethylenediamine tetraacetic acid (EDTA) anticoagulants using sterile technique under fasting conditions. After centrifugation at 2000 *g* for 10 min at 4°C and the plasma collection, the remaining blood has been used to isolate PBMCs according to Boyum methodology (1968) which is based on a gradient density. Tubes with blood and Hank’s solution have been centrifuged at 400 *g* for 25 min at 20°C stopping without brake; PBMCs layer has been recovered and washed twice with cold Hank’s solution by two centrifugation steps at 600 *g* for 10 min at 4°C. Cells have been counted and aliquots with a concentration of 3 millions/ml prepared; these aliquots have been centrifuged at 600 *g* for 6 min at 4°C, Hank’s solution has been removed and pellets have been heat shocked by snap freezing in liquid nitrogen and finally stored at -80°C.

### Protein Extraction

Peripheral blood mononuclear cells frozen pellets at a concentration of approximately 10^6^ cells/ml were lysed in 1x Lysis Buffer (20 mM Tris pH 7.2, 1 mM EDTA, 1 mM DTT, 0.1% NP-40), sonicated for 5 min, vortexed briefly, heat shocked twice by snap freezing for 4 min at -80°C and incubated at 37°C for 4 min. The samples were then centrifuged at 13,000 rpm for 20 min at 4°C and protein concentration of the supernatants was determined using the Bradford Biorad Protein Assay.

### Determination of 20S Proteasome Concentration

Twenty S (20S) levels were measured by 20S/26S Proteasome ELISA Kit (Enzo Life Sciences). Cell lysate samples were diluted 1:250 in provided buffer and measurements were performed in duplicates. Optical density was read at 450 nm on Safire II microplate reader (TECAN). The concentration of 20S Proteasome was determined by interpolation from the standard curve derived from the diluted 20S Proteasome standards and then normalized to the total protein amount (% 20S).

### Chymotrypsin-Like Activity (CT-L)

Proteasome activities were measured in freshly-prepared protein extracts. Triplicates of 10 μg total protein, plus an extra reaction with a specific proteasome inhibitor for each sample, were adsorbed onto 96 well black plates. Diluted substrate (LLVY, UBPBio, Aurora, CO, United States) was then added in a final concentration of 25 μM. The specificity of the reaction was assessed measuring the fluorescence released in the presence of 20 μM MG132 (the proteasome inhibitor). Fluorescence was measured by multiple reads for 60 min at 37°C by TECAN Kinetic Analysis (excitation 380 nm, emission 460 nm, read interval 5 min), following linear regression analysis via Prism Graph Pad 5.0 Software, in which activity was expressed as rfu/min.

### Determination of Protein Carbonyls (Oxidized Proteins)

Carbonyl groups of proteins were detected using the “OxiSelectTM Protein Carbonyl ELISA Kit” (STA-310, Cell Biolabs, Inc., San Diego, CA, United States). Measurements were performed in duplicates and cell lysate samples were diluted ∼250-fold to 10 μg/ml protein with PBS, prior to adsorption onto 96-well Protein Binding ELISA Plates. Absorbance was measured using Safire II microplate reader (TECAN) and the protein carbonyl content in unknown samples was determined using the standard curve derived from the BSA standards.

### Immunoproteasome Composition

Beta 1i immunosubunit (β1i) was revealed via Western Blot analysis of the protein extracts from both subgroups (before and after the intervention). Protein content of cell lysates was measured using Bradford Biorad Protein Assay. Proteins (20 μg) were separated by dodecyl sulfate-polyacrylamide gel electrophoresis and transferred to nitrocellulose membranes for probing. Membranes were incubated overnight with antibodies against β1i subunit (1:1000, Enzo) at 4°C shaking in a 5% non-fat dry milk/0.1% Tween20 TBS solution (1xTBS-T) and were subsequently rinsed three times in 1xTBS-T. One-hour incubation with Goat anti-rabbit secondary antibodies (1:2000, SantaCruz) was followed by three rinses in 1xTBS-T. The bound antibodies were then detected by developing the film in a dark room. The membranes were stripped of antibodies and were re-probed in like manner with a GAPDH (1:4000, 1 h, Sigma) antibody to ensure equal protein loading. The quantification was performed using Image Studio Lite (Version 5.2).

### Statistical Analysis

Statistical analysis was performed using Prism Graph Pad 5.0 Software. Significance was taken as follows: *p*-values < 0.05 (^∗^), *p*-values < 0.01 (^∗∗^), *p*-values < 0.001 (^∗∗∗^), and *p*-values < 0.0001 (^∗∗∗∗^). Positive or negative correlations between the aforementioned biomarkers within the same timeframe (T0 – baseline values for subjects before the intervention or T1 – values for subjects post-intervention) were determined in the total of 120 samples using paired *t*-test with Pearson’s correlation coefficient (*r*). Biomarkers from T0 samples along with corresponding values from T1 samples for distinct subgroups, were analyzed by two-tailed Student’s paired *t*-test. Differences between distinct subgroups for each of the examined biomarkers in T0 or T1 periods were evaluated using unpaired *t*-test. Experimental data were additionally analyzed in association with a number of qualitative variables, relating to subjects’ health and lifestyle profile and provided from the NU-AGE Database using one-way analysis of variance (ANOVA).

## Results

**FIGURE 1:**
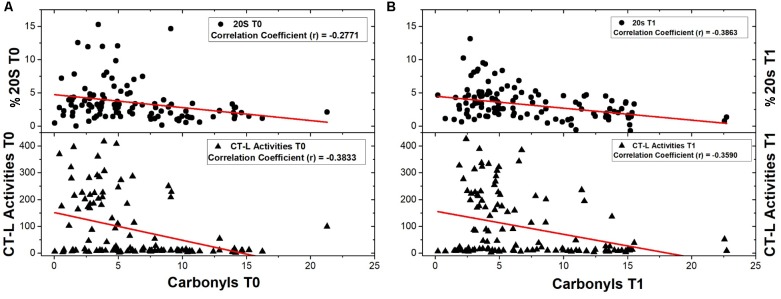
Correlations of 20S content and CT-L activity with protein carbonyls’ levels were examined before and after the intervention as well. **(A)** 20S content and CT-L activity were correlated with protein carbonyls’ levels before any intervention (number of samples: 108 and 119 respectively). **(B)** Analysis of correlations of 20S content and CT-L activity with protein carbonyls’ levels was also performed after the intervention (number of samples: 114 and 115 respectively).

First, a comparative analysis was performed on the data obtained from the total of 120 samples for each biomarker in order to reveal possible interrelations, pre- and post-intervention (T0, T1). As shown in **Figure 1**, a negative correlation was found between proteasome activity and oxidized/carbonylated proteins’ levels both before and after the intervention.

**FIGURE 2:**
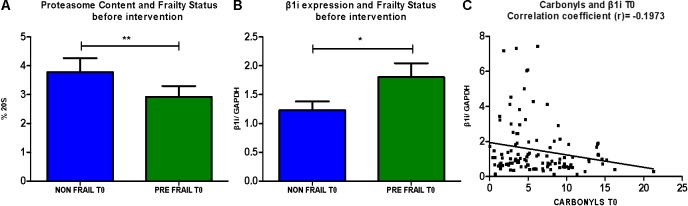
**(A)** Comparative analysis of % of 20S levels between non-frail and pre- frail subjects before the intervention (unpaired *t*-test, Mean ± SEM). **(B)** Comparative analysis of β1i expression between non- frail and pre- frail subjects before the intervention (unpaired *t*-test, Mean ± SEM). **(C)** Analysis of correlation between β1i expression levels and protein carbonyls’ levels before the intervention (paired *t*-test, number of pairs: 115). ^∗^*p* < 0.05 and ^∗∗^*p* < 0.01.

Next we examined the proteasome content (20S) before the intervention. As shown in **Figure 2**, non-frail subjects (*n* = 50) are showing significantly higher levels of 20S in comparison with pre frail subjects (*n* = 29). Moreover, analysis of β1i demonstrated higher expression levels in subjects described as pre frail before intervention (*n* = 31) in comparison with non-frail subjects (*n* = 56). This trend was not observed at T1 time point. Accordingly, β1i levels before the dietary intervention display a negative correlation with oxidized/carbonylated proteins’ levels.

**FIGURE 3:**
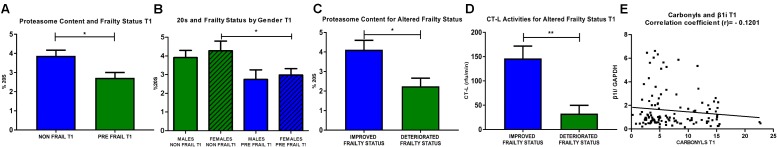
**(A)** 20S proteasome levels after the intervention were analyzed in sub-clusters of non-frail and pre-frail subjects (unpaired *t*-test, Mean ± SEM). **(B)** 20S proteasome levels of non-frail and pre-frail subjects were subsequently analyzed for male and female subjects after the intervention (unpaired *t*-test, Mean ± SEM). **(C)** 20S proteasome levels were determined in subjects that demonstrate improvement in their frailty status after the intervention (from pre-frail at T0 to non-frail at T1) in comparison with the subgroup of deteriorated frailty status (from non-frail at T0 to pre-frail at T1) (unpaired *t*-test, Mean ± SEM). **(D)** Subjects that improved their frailty status were compared with those of the subgroup with deteriorated frailty status, and after intervention, showed significantly increased levels of CT-L activity (unpaired *t*-test, Mean ± SEM). **(E)** Analysis of correlation between β1i expression levels and protein carbonyls’ levels after the intervention (paired *t*-test, number of pairs: 119). ^∗^*p* < 0.05 and ^∗∗^*p* < 0.01.

Next, different clusters, such as males/females and non-frail/pre-frail subjects that either retained or altered their frailty status during the intervention were analyzed in association with each of the examined biomarkers (**Figure 3**). Specifically, regarding the proteasome content (20S) after the intervention, non-frail subjects (*n* = 77) are showing significantly higher levels of 20S in comparison with pre frail subjects (*n* = 42). Subsequent sex analysis revealed a more profound effect in female non-frail subjects (*n* = 36) when compared with female pre frail subjects (*n* = 28). Subjects with improved frailty status (from pre-frail at T0 to non-frail at T1) after the intervention (*n* = 21) retain higher levels of 20S in comparison with the subgroup with deteriorated frailty status (*n* = 12) after the intervention (from non-frail at T0 to pre-frail at T1). As for proteasome activity (CT-L), subjects with improved frailty status (*n* = 21) showed significantly increased levels of CT-L activity in comparison with those characterized by deteriorated frailty status (*n* = 12) after intervention. Moreover, we have observed an inverse association between β1i levels and oxidized/carbonylated proteins’ levels at T1 time-point, similar with that described at T0.

**FIGURE 4:**
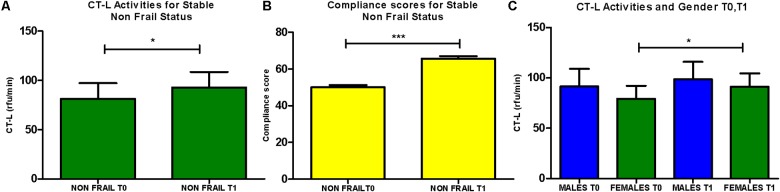
**(A)** CT-L activity levels were analyzed for the subgroup of subjects retaining non-frail status before after the intervention (*n* = 76; paired *t*-test, Mean ± SEM). **(B)** Compliance to the Mediterranean diet of subjects retaining non-frail status before after the intervention (*n* = 76; paired *t*-test, Mean ± SEM). **(C)** CT-L activity levels were determined in the subgroups of males and females before and after intervention (*n* = 51, *n* = 68, respectively, paired *t*-test, Mean ± SEM). ^∗^*p* < 0.05 and ^∗∗∗^*p* < 0.001.

In addition, subjects that retained their non-frail status after the intervention (*n* = 76) showed a significant increase of CT-L activity, while a similar trend for the same subgroup was also observed in the recorded levels of compliance to MedDiet. Interestingly, female subjects (*n* = 68) receive greater benefit from the intervention comparing to the response of male subjects, as they are showing in total significantly increased levels of CT-L activity (**Figure 4**). Finally, the carbonylated protein levels showed no significant differences before and after intervention (data not shown).

## Discussion

This study has evaluated the putative beneficial effects of a specific intervention with dietary-derived compounds from the Mediterranean area, namely the NU-AGE diet (Berendsen et al., 2014). The intervention was hence assessed with emphasis on its proteostasis efficiency. A summary of our findings is depicted in **Table 1**.

**Table 1:** Descriptive statistics of interrelations with statistical significance among different variables, concerning obtained experimental data and specific characteristics of the examined population.

Interrelations among variables	Statistical test	*p*-value	Correlation coefficient (*r*)
% 20S content and protein carbonyls’ levels	Spearman correlation	^∗∗∗^	-0.3863
CT-L activity and protein carbonyls levels	Spearman correlation	^∗∗∗^	-0.3590
% 20S content and age	Spearman correlation	^∗∗∗^	-0.3103
β1i expression and protein carbonyls’ levels in T0	Spearman correlation	^∗^	-0.1973
β1i expression and protein carbonyls’ levels in T1	Spearman correlation	^∗^	-0.1201
CT-L activity and β1i in T0	Spearman correlation	^∗∗∗^	0.3055
β1i expression and compliance in T1 in non-frail subjects	Spearman correlation	^∗∗∗^	-0.0903
CT-L activity and % 20S in T1	Spearman correlation	^∗∗∗^	0.4358
% 20S content increase in non-frail subjects compared with pre-frail	Unpaired *t*-test	^∗^	N/A
% 20S content increase in non-frail female subjects compared with pre-frail	Unpaired *t*-test	^∗^	N/A
% 20S content increase in subjects that improved their frailty status	Unpaired *t*-test	^∗^	N/A
Higher levels of β1i in pre frail subjects than in non-frail	Unpaired *t*-test	^∗^	N/A
CT-L activity increase in tested non-frail subjects	Paired *t*-test	^∗^	N/A
CT-L activity increase in female subjects	Paired *t*-test	^∗^	N/A
Compliance to MedDiet increase in non-frail subjects	Paired *t*-test	^∗∗∗^	N/A
β1i expression increased in subjects with BMI > 30 in T0	One way ANOVA	^∗^	N/A

*^∗^p < 0.05 and ^∗∗∗^p < 0.001.*

The gradually declining efficiency of proteostasis during aging has been proposed as a major underpinning mechanism in common age-related human disorders (Morimoto and Cuervo, 2014; Chondrogianni et al., 2015b). Numerous *in vitro* and *in vivo* studies support a strong correlation between the maintenance of proteostasis and healthy aging. For instance, our previous work has indicated that centenarians have an unremitting expression of functional proteasome which may contribute to their successful aging (Chondrogianni et al., 2000). Accordingly, we have previously demonstrated that “proteasome activated” cell lines exhibit a significant delay of senescence (Catalgol et al., 2009), while a wide range of natural compounds, many of whom are commonly encountered in the Mediterranean diet, have been identified as enhancers of proteasome activity and retarders of aging (Chondrogianni et al., 2010; Kapeta et al., 2010; Papaevgeniou et al., 2016). Furthermore a continuously increasing epidemiological evidence supports the prominent negative role of oxidative stress in aging, cancer, atherosclerosis and cardiovascular disease, age-related diseases and mortality (Monda et al., 2014; Weber et al., 2017; Tosti et al., 2018) as well as in several medical conditions, such as neurodegenerative diseases, obesity, or diabetes mellitus (Greilberger et al., 2008; Mehdi et al., 2013). Fairly common conditions such as overnutrition can induce increased accumulation of neutral lipids and a series of cytotoxic effects associated with impaired redox homeostasis and apoptotic mechanisms. Short term low-fat dietary intervention along with moderate physical activity have been proved to ameliorate oxidative/antioxidative status, from the perspective of biochemical biomarkers examination pre/post intervention. Furthermore, this type of intervention was related with induced alterations in the expression of molecules involved in pathways associated with ER stress and cell death, such as Hsp27 and ERK kinases, demonstrating its protective effect against pro-oxidative, hepatotoxic events (Monda et al., 2014). All those data further support the protective role of certain antioxidants, nutrients, and natural compounds (Stuetz et al., 2016). In contrast, insufficient antioxidant protection can lead to oxidative stress induced damage, with an occurring enhanced formation of oxidative stress biomarkers (Burkle et al., 2015), such as levels of carbonylated proteins. As shown recently, low intake of dietary antioxidants may result in modifications in lipoprotein oxidation and thus increase the risk of developing atherosclerotic plaques (Tosti et al., 2018). Findings presented in this study highlight a significant negative correlation between levels of oxidized/carbonylated proteins and proper proteasomal function both before and after intervention.

Some of the major outcomes of this study refer to increased proteasome content levels, reported in distinct subgroups, such as the one consisting of subjects that retained their non-frail status throughout the intervention period and women. Regarding specifically the proteasome activity, women seem to receive greater benefit, as elevated levels were described in female subjects as well as in the subgroup of consistently non-frail subjects. This increase interestingly demonstrates similar trends with the overall compliance to MedDiet and the achievement of dietary goals.

In order to elucidate subjects’ inflammatory status, an additional biomarker, namely the immunoproteasome, was determined before and after the dietary intervention. The role of the immunoproteasome have been most extensively investigated in the context of inflammatory and autoimmune diseases. Specifically, elevated immunoproteasome levels have been reported in a number of inflammatory and autoimmune diseases, such as ulcerative colitis (Basler et al., 2010), Crohn’s disease (Visekruna et al., 2009), inflammatory bowel disease (IBD) (Fitzpatrick et al., 2007) and hepatitis (Vasuri et al., 2010). These findings clearly support the involvement of the immunoproteasome in these inflammatory and autoimmune diseases and specifically, upregulation of the β1i and β5i subunits has been observed in animal models of neurodegenerative diseases, such as Alzheimer’s disease, Huntington’s disease, and amyotrophic lateral sclerosis (Wagner et al., 2017). However, the specific functions carried out by the immunoproteasome are not fully understood in aging and neurodegenerative diseases and the possible therapeutic implications require further exploration. In this study, negative correlation with oxidized/carbonylated proteins’ levels was demonstrated more prominently before the dietary intervention, along with relatively low levels of proteasome content, proposing that the immunoproteasome may compensate for impaired function of constitutive proteasomes (Niewerth et al., 2014). The produced data also imply that the immunoproteasome is largely involved in the aging process, remarking that β1i expression is related to an increased frailty status with subjects described as pre frail before intervention demonstrating higher β1i expression in comparison with non-frail subjects. This trend was not observed after the intervention, where levels seem to be stabilized, suggesting a potentially beneficial effect of the “NU-AGE diet” on cellular immunophenotype. In support, in non- frail subjects after the intervention, a negative correlation was assessed between β1i expression levels and subjects’ compliance to MedDiet. Interestingly, lower levels of β1i before the intervention were also detected in subjects with lower BMI.

It is also worth mentioning that the examined biomarkers were additionally analyzed in association with qualitative variables related with subjects’ nutritional habits, their pharmacological profile along with levels of fatigue and sleep habits (one way ANOVA; data non-shown). Some preliminary correlations were observed. For instance, proteasome levels were found significantly lower in obese subjects, while immunoproteasome levels correlate negatively. These data further support the notion that features such as high BMI associate with the aging process (Díaz-Ruiz et al., 2015). Overall, the observed trends provided some first insights about the beneficial effect of the used intervention.

## Conclusion

This study provides evidence that adherence to a Mediterranean diet may enhance proteasome and immunoproteasome activity and content in the elderly. Changes that occurred only in either of the subgroups suggest the existence of features, like genetic and epigenetic factors, that can influence the potency of dietary interventions and produce, thus, efficacy variations. Hence, the various proteasome complexes and their substrates, represent effective targets of intervention.

## Author Contributions

EG, CFr, AS, NC, and BP contributed in the design of the conceptual framework. AS, CFa, BP, and JK were in charge of sample collection, preparation, and distribution. SA, VD, and KV carried out the experiments in NHRF. SA and KA conducted the statistical analysis. SA wrote the manuscript with critical feedback and inputs from EG, CFr, AS, NC, and BP.

## Conflict of Interest Statement

The authors declare that the research was conducted in the absence of any commercial or financial relationships that could be construed as a potential conflict of interest.

## Abbreviations

BMI: body mass index
CT-L: chymotrypsin- like proteasomal catalytic activity
GAPDH: glyceraldehyde 3-phosphate dehydrogenase
Inflammaging: low grade inflammatory status in the elderly
N/A: non-applicable
PBMCs: peripheral blood mononuclear cells
Proteostasis: protein homeostasis
T0: timeframe before the intervention
T1: timeframe after the intervention
UPS: ubiquitin-proteasome system
% 20S: percentage of proteasome content in total protein extracts
β1i: beta- 1 subunit of the immunoproteasome.
